# Marchiafava-Bignami Type A Disease: A Rare Neurological Manifestation in Oropharyngeal Cancer

**DOI:** 10.7759/cureus.75308

**Published:** 2024-12-08

**Authors:** Rafaela Lopes Freitas, Mariana Quelhas, Adelina Pereira

**Affiliations:** 1 Internal Medicine Service, Pedro Hispano Hospital - Matosinhos Local Health Unit, Matosinhos, PRT; 2 Internal Medicine Service, Unidade Local de Saúde do Norte Alentejano, Elvas, PRT; 3 Internal Medicine Service, Pedro Hispano Hospital - Matosinhos Local Health Unit, Matosinhos, Porto, PRT

**Keywords:** corpus callosum demyelination, esophageal cancer, malnutrition, marchiafava-bignami disease, thiamine deficiency

## Abstract

Marchiafava-Bignami disease (MBD) is a rare condition characterized by demyelination and necrosis of the corpus callosum, most commonly associated with chronic alcohol consumption. However, it can also occur in non-alcoholic patients and may present secondary to other underlying conditions. We report a case of a 52-year-old male with no history of alcohol use or significant comorbidities, presenting with impaired consciousness and severe malnutrition. Neuroimaging findings confirmed MBD, and further investigation revealed an underlying oropharyngeal malignancy that likely precipitated the disease through feeding difficulties and nutritional deficits. Analytical findings revealed severe metabolic derangements, including hypoalbuminemia and vitamin deficiencies. Despite aggressive treatment, the patient succumbed to his condition. This case highlights the importance of considering MBD in the differential diagnosis of neurological dysfunction in patients with significant malnutrition and emphasizes the need for a thorough investigation into underlying causes, including malignancy.

## Introduction

Marchiafava-Bignami disease (MBD) is a rare and potentially fatal neurological condition, primarily associated with chronic alcohol consumption [1], characterized by demyelination and necrosis of the corpus callosum. However, it has also been reported in non-drinkers, with malnutrition being a key factor [2].

The clinical presentation of MBD is highly variable, ranging from mild cognitive impairment to profound neurological deficits, including altered consciousness, dysarthria, and gait disturbances [3].

In recent years, an expanding body of literature has described MBD in association with underlying conditions unrelated to alcohol, such as malignancies, severe infections, and gastrointestinal diseases.

These cases emphasize the importance of identifying and treating precipitating factors to improve outcomes. We present a unique case of MBD in a middle-aged male, which was the first manifestation of an underlying oropharyngeal cancer.

## Case presentation

A 52-year-old male with no prior relevant medical history was admitted to the emergency department with altered mental status, dyspnea, and dysphagia.

Upon initial evaluation, the patient had audible stridor and an oxygen saturation of 88% (Fi 0.21) requiring supplemental oxygen via a non-rebreather mask. Neurological examination revealed a Glasgow Coma Scale (GCS) score of 10 (eye: 3, verbal: 3, motor: 4) with poor responsiveness to verbal commands. Additional findings included horizontal nystagmus and dysarthria.

The patient exhibited severe signs of malnutrition (stage 2 of the Global Leadership Initiative on Malnutrition - GLIM criteria) including >10% of weight loss in less than six months, a body mass index of 16 kg/m², muscle wasting, and hypoalbuminemia (2.1 g/dL).

Laboratory investigations revealed hyponatremia (134 mmol/L), hypokalemia (3.2 mmol/L), hypocalcemia (7.6 mg/dL), and hypomagnesemia (1.3 mg/dL). Vitamin assessments showed severe thiamine deficiency (<10 µg/dL) and low levels of vitamin B12 (100 pg/mL) and folates (1.5 ng/mL) (Table 1).

**Table 1:** Analytical findings.

Parameter	Value	Reference range
Hemoglobin	12.2	13-17.0 g/dL
White blood cell count	12.1	4-11.0 /uL
Platelet count	150.000	150-450.000 /uL
Serum Albumin	2.1	3.5-5.0 g/dL
Sodium	134	135-145 mmol/L
Potassium	3.2	3.5-5.0 mmol/L
Calcium	7.6	8.5-10.5 mg/dL
Magnesium	1.3	1.7-2.2 mg/dL
Cobalamin (B12 vitamin)	100	189-883 pg/mL
Folic acid (B9 vitamin)	1.5	2.4-18.9 ng/mL
Thiamine (B1 vitamin)	< 10	20-50 ug/dL
C-reactive protein	9	<10 mg/dL
Blood urea nitrogen	45	18-55 mg/dL
Creatinine	0.6	0.7-1.3 mg/dL

**Figure 1:**
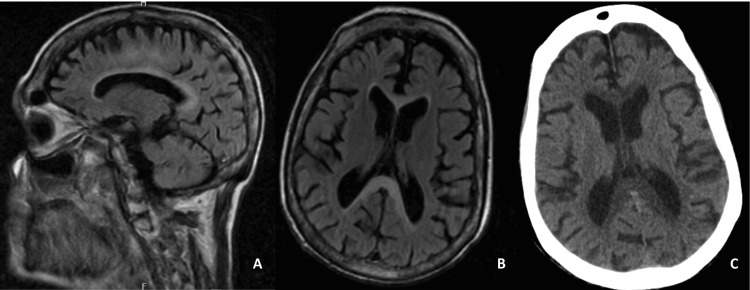
Patient MRI and CT findings. Sagittal (A) and axial (B) FLAIR images showing areas of hyperintensity throughout the corpus callosum. (C) Axial head CT images showing corpus callosum hypodensity.

A cranial computed tomography (CT) scan revealed hypodensity involving the entire corpus callosum (Figure 1A). Subsequent magnetic resonance imaging (MRI) confirmed corpus callosum hyperintensity, predominantly affecting the splenium, as well as T2 hyperintensity in the white matter of the corona radiata and centrum semiovale (Figures 1B, 1C). These findings were consistent with MBD type A.

Cervical CT imaging identified an ulcerative-vegetative neoplasm centered at the vallecular, with infiltration into the epiglottis and tongue base, suggestive of advanced oropharyngeal cancer (T4 Nx Mx).

The diagnosis of MBD was attributed to severe malnutrition and feeding difficulties secondary to the malignant process. The patient was managed with aggressive fluid resuscitation and high-dose intravenous thiamine replacement. Despite these interventions, the patient's condition deteriorated, and he succumbed to his illness one week after admission.

## Discussion

MBD remains a diagnostic challenge due to its rarity and nonspecific clinical presentation [4]. It has no ethnic, racial, or geographic predilection, but there is a higher incidence in men.

MDB pathophysiology is unclear. While it is classically linked to chronic alcohol consumption [5,6], this case underscores the growing recognition of non-alcoholic MBD, particularly in the context of malnutrition and secondary metabolic disturbances [7,8]. Malnutrition in patients with oropharyngeal or esophageal malignancies is often multifactorial, resulting from mechanical obstruction, dysphagia, and cancer-related cachexia. In this case, the neoplasm likely led to a profound nutritional deficit, including thiamine deficiency, which precipitated MBD.

MBD is traditionally divided into two types based on the severity and extent of corpus callosum involvement [3,9].

Type A is the most common and severe form of MBD, characterized by the involvement of the entire corpus callosum, with a predilection for the splenium. Histopathological findings in type A show widespread demyelination and necrosis. MRI typically reveals hyperintensities across the corpus callosum and adjacent white matter, as seen in our patient. This form is often associated with more severe clinical outcomes, including cognitive decline, motor disturbances, and, in many cases, death, especially when the underlying cause is not addressed promptly.

On the other hand, type B involves only partial damage to the corpus callosum, and the clinical presentation is often less severe compared to Type A. Type B may involve milder cognitive and motor deficits, and the prognosis is somewhat better if the underlying condition is treated. MRI findings in type B typically show isolated or limited hyperintensities in the splenium or anterior portion of the corpus callosum.

Our patient displayed classic features of type A MBD with full involvement of the corpus callosum, particularly the splenium, as evidenced by MRI findings.

Neuroimaging remains the gold standard for diagnosing MBD, with MRI providing the most detailed visualization of the corpus callosum changes [10,11]. The characteristic findings include T2 hyperintensities within the corpus callosum and adjacent white matter, as seen in our case.

MRI also helps differentiate MBD from other conditions that might present with similar symptoms, such as multiple sclerosis or acute encephalitis, both of which can also involve white matter. In this case, the rapid onset of neurological symptoms, combined with the patient's malnutrition and underlying malignancy, guided the diagnosis.

Despite early recognition and treatment with high-dose intravenous thiamine and electrolyte management, the prognosis for MBD in patients with advanced malignancy remains poor [2]. In fact, mortality rates are particularly high in cases where MBD is precipitated by conditions like cancer, where treatment options may be limited, and the nutritional deficits are often severe and difficult to correct [9].

This case highlights the importance of considering MBD in the differential diagnosis when faced with unexplained neurological dysfunction in malnourished patients. A thorough diagnostic workup to identify underlying conditions, such as malignancy, infections, or metabolic disturbances, can help guide effective treatment and improve outcomes. Early recognition and treatment of thiamine deficiency, along with appropriate management of the precipitating factor (e.g., cancer treatment), are crucial in preventing further neurological deterioration.

## Conclusions

MBD is a rare but severe neurological disorder that can occur in non-alcoholic patients with severe malnutrition. This case emphasizes the importance of considering MBD in the differential diagnosis of altered consciousness in malnourished individuals and highlights the potential role of underlying malignancies as precipitating factors.

Early diagnosis and aggressive treatment are critical, although the prognosis remains guarded in advanced cases. Further research is needed to better understand the pathophysiology and optimal management strategies for non-alcoholic MBD.
